# Antibody Response against Circulating Omicron Variants 8 Months after the Third Dose of mRNA Vaccine

**DOI:** 10.3390/vaccines10091512

**Published:** 2022-09-10

**Authors:** Gabriele Anichini, Chiara Terrosi, Gianni Gori Savellini, Claudia Gandolfo, Francesca Barbagli, Giulio Alberto Carta, Simonetta Fabrizi, Giovanni Battista Miceli, Maria Grazia Cusi

**Affiliations:** 1 Virology Unit, Department of Medical Biotechnologies, University of Siena, 53100 Siena, Italy; 2 Virology Unit, Santa Maria AlleScotte University Hospital, V.le Bracci 1, 53100 Siena, Italy; 3 Preventive Medicine and Health Surveillance Unit, Santa Maria AlleScotte University Hospital, V.le Bracci 1, 53100 Siena, Italy

**Keywords:** SARS-CoV-2, Omicron, BA.2, BA.5, mRNA vaccine, BNT162b2, mRNA-1273

## Abstract

The COVID-19 wave is being recently propelled by BA.2 and, particularly, BA.5 lineages, showing clear transmission advantages over the previously circulating strains. In this study, neutralizing antibody responses against SARS-CoV-2 Wild-Type, BA.2 and BA.5 Omicron sublineages were evaluated among vaccinees, uninfected or infected with Omicron BA.1 strain, 8 months after the third dose of SARS-CoV-2 vaccine. The aim of this study was to compare the cross-protective humoral response to the currently circulating variant strains induced by vaccination, followed by Omicron infection in some subjects. Results showed a low antibody titer against all three variants in uninfected vaccinated subjects. On the other hand, vaccinated subjects, infected with BA.1 variant after receiving the third dose (about 40 days later), showed a strong response against both BA.2 and BA.5 strains, albeit with lower titers. This reinforces the concept that vaccination is fundamental to induce an adequate and protective immune response against SARS-CoV-2, but needs to be updated, in order to also widen the range of action towards emerging variants, phylogenetically distant from the Wuhan strain, against which the current formulation is targeted.

## 1. Introduction

The SARS-CoV-2 Omicron (B.1.1.529) variant, first detected in Botswana and South Africa in November 2021, is characterized by a higher transmissibility than the other SARS-CoV-2 variants [1,2]. This phenomenon is strictly related to the high number of mutations present throughout the entire genome, especially in the Spike protein [2], which makes the virus able to partially evade the pre-existing protective antibody response of the host [3,4,5,6,7,8,9,10,11,12]. However, symptoms are mild, compared to those caused by previous variants [13,14,15], especially in light of the reduced neutralizing antibody (NTAb) response over time in vaccinees [16,17,18]. So far, at least five major Omicron sublineages have been identified: BA.1, BA.2, BA.3, BA.4 and BA.5. In particular, the COVID-19 wave is being recently propelled by BA.2 and, particularly, BA.5 lineages, showing clear transmission advantages over the previously circulating viruses [19,20].

In this study, neutralizing antibody responses against SARS-CoV-2 Wild-Type, BA.2 and BA.5 Omicron sublineages were evaluated among vaccinees, uninfected or infected with the Omicron BA.1 strain. The aim of this study was to compare the cross-protective humoral response to the currently circulating variant strains induced by vaccination, followed by Omicron infection in some subjects.

## 2. Materials and Methods

### 2.1. Study Design and Participants

In this observational cohort study, 100 healthcare workers from ‘Santa Maria alleScotte’ University Hospital in Siena (31 males, 69 females; mean age 43.9 years, range 25–63), who had received a third dose of mRNA-1273 (Moderna) vaccine after a two-dose cycle of BNT162b2 vaccine (Pfizer Inc., New York, NY, USA), approximately eight months before testing (average time: 230.4 days, range 215–247), were enrolled. Fifty of them (fourteenmales, thirty-six females; mean age 41.7 years, range 25–62) had a past COVID-19 diagnosis, confirmed both by PCR and serological testing (between December 2021 and January 2022, approximately 40 days after the third dose of vaccine). Sequencing revealed that all subjects had been infected with the Omicron BA.1 strain.

The remaining 50 subjects (16 males and 34 females; mean age 46.3 years, range 26–63) were never infected. However, among them, 14 out of 50 (26%) were infected about 15 days after sampling. All subjects were from the Siena area. Those infected with SARS-CoV-2 showed mild symptoms, such as fever, weakness, rhinorrhea for 7–10 days or no symptoms.

In order to evaluate the humoral response induced by the vaccine, a blood sample was drawn from all the subjects to detect specific IgG against SARS-CoV-2 spike receptor-binding domain (RBD). Moreover, all selected subjects’ sera were tested for the presence of specific neutralizing antibodies against the Wild-Type strain, BA.2 and BA.5 Omicron variants.

The date of the first SARS-CoV-2-positive swab was assumed to be the first day of infection for the sake of simplicity. This research was carried out according to the principles of the Helsinki declaration, with reference to BIOBANK-MIU-2010 document approved by the Ethics Committee with amendment No. 1, on 17 February 2020. Prior to participating in this study, all subjects signed a written informed consent form.

### 2.2. SARS-CoV-2 IgG Antibody Detection

Subjects’ sera were analyzed using Abbott SARS-CoV-2 IgGII Quant assay (Abbott Laboratories, Chicago, IL, USA), a chemiluminescent microparticle immunoassay (CMIA) to evaluate the immune status of individuals with quantitative measurement of IgG antibodies against the spike receptor-binding domain (RBD) of SARS-CoV-2. This assay was performed on an Abbott Alinity (Abbott Diagnostics) according to the manufacturer’s instructions. A sample was considered positive when the result was >50.0 AU/mL. Values higher than 40,000 AU/mL were not investigated further and reported as 40,000, as it was the limit of the kit detection.

SARS-CoV-2 natural infection was confirmed by using Abbott SARS-CoV-2 anti-nucleocapsid IgM and IgG assay (CMIA), according to the manufacturer’s instructions. The final interpretation of positivity wasdetermined by a ratio above a threshold value, ≥1.4 relative light units (RLU).

### 2.3. SARS-CoV-2 Microneutralization Test

SARS-CoV-2 virus neutralization assay was carried out on Vero E6 cells in a 96-well microplate. Twenty-five microliters of two-fold serial dilutions (1:8 to 1:1024) of sera samples were added to an equal volume of SARS-CoV-2 Wild-Type (WT) (SARS-CoV-2/human/ITA/Siena-1/2020; GenBank:MT531537.2), Omicron BA.2 (SARS-CoV-2/human/ITA/TUS-Siena-0436626/2022; GenBank:ON974845.1) and BA.5 (SARS-CoV-2/human/ITA/TUS -Siena-0435435/2022; GenBank:ON974846.1) containing 100 TCID_50_ and incubated for 90 min at 37 °C. Finally, 50 μL of Vero E6 cell suspension (2 × 10^5^ cells/mL) prepared in complete DMEM were added to each well. After incubation at 37 °C, cultureswere examined daily for the presence of CPE under microscope (Olympus IX51). The 50% end-point titer was calculated by using the Reed–Muench method [21]. Positive and negative control sera were included in each assay [22]. Geometric mean titers (GMTs) of the neutralization assays were calculated. Values higher than 1:1024 were not investigated further.

### 2.4. Statistical Analysis

Differences among age, circulating IgG levels, and neutralizing geometric mean titers (GMTs) were evaluated and statistical significances were assessed with a two-tailed chi-squared test. Results were considered statistically significant at *p* < 0.05. For each variable, 95% confidence interval (CI 95%) was calculated and reported. All analyses were performed by using GraphPad Prism 7.0 software.

## 3. Results

**Figure 1:**
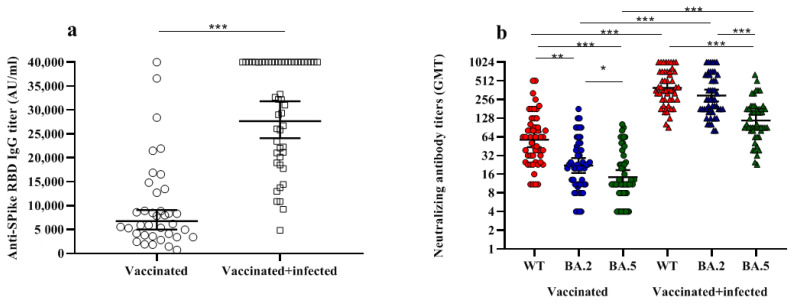
Immune response in vaccinated subjects, either infected with SARS-CoV-2 BA.1 variant or not. Anti-spike IgG antibody levels (Panel (**a**)) and neutralizing SARS-CoV-2 antibodies (Panel (**b**)) in serum samples of vaccinated subjects with three doses of mRNA vaccine, either infected (squares) or not (circles) with Omicron BA.1 strain. Differences in neutralizing IgG antibodies were evaluated against WT (red), BA.2 (blue), and BA.5 (green) strains (Panel (**b**)). In each plot, the horizontal line represents the mean (Panel (**a**)) or the geometric mean (Panel (**b**)), while the top and bottom lines show the 95% confidence interval (CI 95%). The *p* values are reported in the figures, where * stands for *p* ≤ 0.05, ** for 0.05< *p* <0.001 and *** stands for *p* < 0.001.

Circulating anti-spike IgG against the Wuhan strain (WT) was compared among the two groups of subjects (Figure 1a). Results showed significantly higher levels of circulating IgG in infected vaccinees than in non-infected vaccinees (mean titers 30,415.2 vs. 9819.2 AU/mL; CI 95% 12,935.0–6702.0 vs. 33,529.0–27,302.0).

In order to compare the cross-protective humoral response against the WT and Omicron BA.2 and BA.5 variants induced by vaccination, we analyzed the neutralizing antibody response in vaccinees and in vaccinated subjects infected with the BA.1 variant (Figure 1b) using a live-virus-based assay [8,18,22]. Results showed a significant difference in the neutralizing antibody response among BA.1-infected and non-infected vaccinated subjects against the WT (GMT = 394.4 vs. 57.9; CI 95% 476.8–326.2 vs. 76.2–43.9), BA.2 (GMT = 296.9 vs. 22.2; CI 95% 369.2–238.7 vs. 29.2–16.8), and BA.5 variants (GMT = 117.4 vs. 14.3; CI 95% 145.6–94.7 vs. 18.7–11.0) (*p* < 0.001 for all three strains). The data confirmed the booster effect of Omicron infection on the vaccinees, regardless of the variant tested in the neutralization assay (Figure 1b). Moreover, we observed that BA.1 infection, which induced a strong and comparable response in vaccinated subjects against both WT and BA.2 strains (GMT = 394.4 and 296.9, *p* > 0.5), induced a significantly lower response against BA.5 (GMT = 117.4, *p* < 0.0001). On the contrary, the protective response in the vaccinees highlighted a low immune responsiveness, induced by the currently administered vaccine against all Omicron sublineages, when tested against BA.2 (GMT = 57.9 vs. 14.3, *p* < 0.001) and BA.5 (GMT = 14.3 vs. 22.2, *p* = 0.04).

Finally, naïve vaccinated subjects, who had been infected about 15 days after sampling (13/50), showed a neutralizing titer against all strains comparable to that of the uninfected group (GMT WT = 48.1 vs. 62.5; BA.2 = 17.5 vs. 23.0; BA.5 15.2 vs. 14.8; *p* > 0.5). Indeed, GMTs against BA.5 were below the threshold to confer protection to SARS-CoV-2 [23].

## 4. Discussion

The current growth advantage for the BA.5 variant of concern, compared to the previous BA.2 variant, is probably due to its ability to evade immune protection from infection, induced by prior infection or vaccination, particularly if the humoral response has waned over time [8,12]. Indeed, BA.1 Omicron infection alone induces a modest response in the unvaccinated subjects [5], which does not offer protection against the WT or Delta strains.

In the current study, we analyzed the antibody response using achemiluminescent assay (CMIA) and microneutralization assay eight months after the third heterologous booster vaccine in 100 healthcare workers from our hospital. Among them, half had been infected with the BA.1 strain 40 days after the third dose, about seven months before being screened. The other group was represented by subjects vaccinated eight months earlier, who had never been infected. The two groups’ humoral immune response was compared. We analyzed the cross-neutralizing antibody response both against the Wild-Type, in order to assess the response generated by vaccination, and BA.2 and BA.5 variants, these being the last two lineages selected as prevalent circulating strains at the time of sampling.

The results showed a low antibody titer against all three variants in uninfected vaccinated subjects, likely protective against WT, but not against BA.2 and BA.5 variants. However, these titers were comparable to those of unvaccinated subjects infected with BA.1 about 40 days before screening, confirming that natural infection alone induces a low neutralizing response to most variants [5]. On the other hand, vaccinated subjects, infected with the BA.1 strain seven months before, showed a strong response both against the BA.2 strain, phylogenetically close to BA.1, and even the BA.5 strain, albeit with lower titers, due to the presence of three additional mutations in the Spike RBD domain (L452R, F486V, and R493Q reversion) [24]. The third dose of the vaccine was highly effective in preventing COVID-19-associated hospitalizations during both Delta- and Omicron-predominant periods, underscoring the importance of receiving a third dose of mRNA COVID-19 vaccine to prevent both moderately severe and severe COVID-19 [25,26]. Although protection decreases with time, a third dose was still highly effective at preventing severe illness with Omicron [27]. Moreover, hybrid immunity was shown to trigger a very strong antibody responseover an extended period, showing how important vaccination is even for people who have been previously infected with SARS-CoV-2, in order to ensure the most effective protection against COVID-19 [28].

Therefore, an additional dose of the vaccine is needed, although with a different formulation from the current one, as it is poorly protective against Omicron subvariants (GMT = 22.2 against BA.2 and 14.3 against BA.5). Indeed, 13 out of the 50 uninfected vaccinated subjects were infected with the BA.5 variant about 15 days after sampling, thus leading to the assumption that the occurrence of infection was due to the presence of a pre-existing low neutralizing antibody titer, as well as an increased exposure risk to the circulating Omicron variant predominance. Nevertheless, no vaccinated subjects previously infected with BA.1 were reinfected during this short observational period, likely inducing cross-protection against Omicron BA.5 infection, due to the similarity between the two strains. Indeed, rare cases of reinfection have been described so far [29,30].

Even a natural infection with BA.1 alone, without prior vaccination, does not guarantee a high and sustained response, raising doubts about possible protection in case of re-exposure to BA.5 [5,8]. This reinforces the concept that vaccination is fundamental to induce an adequate and protective immune response against SARS-CoV-2, but needs to be updated, in order to also widen the range of action towards emerging variants, phylogenetically distant from the Wuhan strain, against which the current formulation is targeted. This might help to slow the spread of the SARS-CoV-2 virus.

## Data Availability

The data supporting the reported results can be provided by the corresponding author upon reasonable request.
